# Clinicians' and Patients' Experiences and Perceptions on the Prevention and Management of Surgical Site Infections: A Mixed‐Methods Systematic Review

**DOI:** 10.1111/jocn.17443

**Published:** 2024-11-22

**Authors:** Eliza Humphrey, Adam Burston, Elizabeth McInnes, Heilok Cheng, Mika Musgrave‐Takeda, Ching Shan Wan

**Affiliations:** ^1^ School of Nursing, Midwifery and Paramedicine Australian Catholic University Melbourne Victoria Australia; ^2^ School of Nursing, Midwifery and Paramedicine Australian Catholic University Ballarat Victoria Australia; ^3^ School of Nursing, Midwifery and Paramedicine Australian Catholic University Brisbane Queensland Australia; ^4^ Nursing Research and Practice Development Centre The Prince Charles Hospital Chermside Queensland Australia; ^5^ Nursing Research Institute, St Vincent's Health Network Sydney St Vincent's Hospital Melbourne and Australian Catholic University Melbourne Victoria Australia; ^6^ National Health and Medical Research Council Centre of Research Excellence in Wiser Wound Care Griffith University Gold Coast Queensland Australia; ^7^ Respiratory Research @ Alfred, School of Translational Medicine Monash University Melbourne Victoria Australia

**Keywords:** clinicians, evidence‐based practice, experiences, management, patients, preferences, prevention, surgical site infections, systematic review

## Abstract

**Aim:**

To explore clinicians' and patients' perceptions of implementing evidence‐based practice to improve clinical practice for preventing and managing surgical site infections within hospital acute care settings.

**Design:**

A convergent integrated mixed‐methods systematic review using the Joanna Briggs Institute approach.

**Methods:**

Included studies reported (i) acute care hospital clinicians' and patients' experiences and preferences for preventing and managing surgical site infections and (ii) barriers and facilitators to implementing surgical site infection prevention and management guidelines. The Mixed Methods Appraisal Tool and the Quality Improvement Minimum Quality Criteria Set were used for critical appraisal. Quantitative data was transformed into qualitised data, then thematically synthesised with qualitative data and coded all findings into themes. Clinicians' and patients' views were also compared.

**Data Sources:**

English language peer‐reviewed studies published from 2009 to March 2023 were identified from Medline, EMBASE, CINAHL, PsycINFO and Cochrane Central Library.

**Results:**

Thirty‐seven studies (16 quantitative, 17 qualitative, 3 mixed‐methods and 1 quality improvement) met the inclusion criteria. Five main themes represent key factors believed to influence the implementation of evidence‐based surgical site infection prevention and management guidelines: (1) Intentional non‐adherence to insufficiently detailed and outdated guidelines, (2) Knowledge deficits on evidence‐based SSI care bring about inconsistent clinical practice, (3) Collaborative interdisciplinary and patient‐provider relationship to enhance guideline uptake, (4) Infection surveillance to improve patient safety and quality of life and (5) Negative physical and psychological impacts on patients.

**Conclusion:**

The five themes reflect a need for updated hospital guidelines as a medium to improve surgical site infection knowledge and ensure consistent and evidence‐based clinical practice. This review also highlights the significance of interdisciplinary and patient‐provider collaboration and infection surveillance to facilitate guideline uptake. The effectiveness of intervention bundles designed to improve these aspects of care will need to be evaluated in future research.

**Impact:**

A future intervention bundle that includes (1) ensuring up‐to‐date hospital guidelines/policies; (2) fostering collaborative interdisciplinary teamwork culture between physicians, nurses, podiatrists, pharmacists and allied health professionals; (3) encouraging patient or carer involvement in shared decision‐making and (4) implementing audit and feedback mechanism on infection surveillance is proposed to improve SSI prevention and management in acute care settings.

**Reporting Method:**

This paper followed the PRISMA 2020 checklist guideline for reporting systematic reviews.

**Patient or Public Contribution:**

This mixed‐methods systematic review collates evidence of clinicians' and patients' experiences and preferences for preventing and managing surgical site infections. The inclusion of hospital patients' perspectives supports the development of patient‐centred interventions.

**Trial Registration:** The review protocol is registered on the International Prospective Register of Systematic Reviews (PROSPERO 2021 CRD42021250885). Available at: https://www.crd.york.ac.uk/prospero/display_record.php?ID=CRD42021250885

**Summary**

- Implications for the health profession and patient care
  ○ Understanding and addressing barriers to preventing and managing surgical site infections in acute care is crucial in promoting patient safety. These review findings provide new insights into approaches to improve surgical site infection prevention and management. According to our findings, fostering an interdisciplinary work culture and having regular infection surveillance are suggested to improve guideline uptake at the system level. Providing continuous professional education on updated guidelines on preventing and managing surgical site infection and patient‐provider interaction training to health professionals are thought to improve evidence‐based clinical practice. At the patient level, encouraging a proactive attitude and patient involvement in surgical site infection‐related decision‐making is believed to enhance patient‐centred care.
- What does this paper contribute to the wider global clinical community?
  ○ The findings from this paper inform the development of a multifaceted intervention addressing context‐specific barriers linked to updating guidelines, continuous clinical education, interdisciplinary collaboration, patient‐provider shared decision‐making and infection surveillance to improve the prevention and management of surgical site infections in acute care hospitals globally.

## 1 Introduction

Surgical site infection (SSI), defined as an infection that develops in the region of the body where surgery has been performed (Australian Commission on Safety and Quality in Health Care 2018), is indisputably preventable, yet they constitute up to 20% of all healthcare‐associated infections (HAI) (National Institute for Health and Care Excellence 2019). In low‐ and middle‐income countries, SSIs are the most common type of HAI, affecting up to one‐third of patients who have had a surgical procedure (World Health Organisation 2018). SSI also brings a huge economic burden to health services and patients. In the United States, SSIs are the second leading cause of HAI, with an annual financial burden of $3.5–$10 billion (Sen 2019). In Australia, the direct cost of SSIs in public hospitals during 2018–19 was $323.5 million (Royle et al. 2023). SSI‐associated harms to patients include physical and mental health distress, increased mortality rates, delayed wound healing and prolonged length of inpatient stays, which result in increased financial burdens for patients (Australian Commission on Safety and Quality in Health Care 2018; Avsar et al. 2021).

Yet, SSI incidence rates can be as low as 2%–11% globally when appropriate prevention and management strategies are maintained (Berríos‐Torres et al. 2017). The World Health Organisation (2018) established the first‐ever global guidelines for preventing SSIs in 2016, updated in 2018. The guideline recommends 29 ways to prevent SSIs to address the main concern for governments and healthcare providers (World Health Organisation 2023).

Although national and international clinical guidelines on SSIs are widely available (World Health Organisation 2018; National Health and Medical Research Council 2019; National Institute for Health and Care Excellence 2019), SSI rates were not reduced between 2014 and 2019 (Leaper et al. 2014; Zucco et al. 2019). The acute care hospital setting is complex and frequently changing (Figueroa et al. 2019). These emergent issues remain not well understood and encompass individual‐, organisational‐ and system‐level challenges to the guideline uptake (Leaper et al. 2014; Morikane et al. 2021).

Recent studies and trials conducted within acute care hospital settings focusing on reducing SSI incidence by incorporating multitudes of interventions such as optimised statistical process control charts and educating patients on modifiable risk factors have been identified as warranting promise in reducing SSIs (Anderson et al. 2020; Horgan, Hegarty, et al. 2023). However, these interventions are yet to be evaluated as a strategy to prevent SSIs, therefore presenting a gap in the literature. Additionally, recent systematic reviews propose the importance of multifaceted implementation strategies in improving the uptake of SSI guidelines (Ariyo et al. 2019; Tomsic et al. 2020). Theory‐driven multifaceted intervention development using a bottom‐up approach according to the Knowledge to Action Framework has been shown to be more effective than interventions developed without a theoretical basis (Field et al. 2014; Teggart et al. 2022). However, it is unclear from end‐users' views what clinically important and relevant factors influence guideline uptake and what appropriate implementation strategies to include in these multifaceted interventions (Ariyo et al. 2019, Tomsic et al. 2020).

Hospital clinicians are the key end‐users in implementing SSI guidelines. Using a bottom‐up approach to understand clinicians' perspectives on barriers and facilitators to guideline uptake will help identify key elements in the multifaceted intervention (Craig et al. 2017). Understanding patients' preferences and perceived needs in wound management drives clinicians' approach to wound care decision‐making (Gillespie et al. 2014; Heerschap, Nicholas, and Whitehead 2018). Comprehending patients' views and preferences for wound management in the shared decision‐making process is vital to enhancing patient‐centred quality of care and facilitating evidence‐based clinical practice change (Conner et al. 2023). Therefore, understanding clinicians' and patients' views is necessary to identify potential key implementation strategies to facilitate clinical practice change (Pereira et al. 2022; Tomsic et al. 2020; Ariyo et al. 2019).

Even though research has explored clinicians' and patients' views on the prevention and management of SSI, no mixed‐methods systematic review (MMSR) has synthesised this evidence to provide insights into key factors contributing to the uptake of SSI prevention and management guidelines. Therefore, this MMSR aimed to collate evidence on hospital clinicians' and patients' perceptions, experiences and preferences for preventing and managing SSIs and the implementation of evidence‐based practice for preventing and managing SSIs. The purpose of this approach was to explore and compare clinicians' and patients' views on SSI prevention and management in acute care settings to provide insights into factors contributing to improving the uptake of the SSI guidelines. Therefore, this review focused solely on the use of clinical practices to prevent and manage SSI in acute and subacute wards rather than practices in intensive care units and outpatient clinics.

## 2 The Review

The protocol for this MMSR was registered on the International Prospective Register of Systematic Reviews (Wan et al. 2021). The review used the convergent integrated approach outlined in the Joanna Briggs Institute Manual for Evidence Synthesis Handbook (Lizarondo et al. 2020) and the Cochrane Handbook guidelines on qualitative evidence synthesis (Higgins et al. 2021). This MMSR is reported in accordance with the Preferred Reporting Items for Systematic Reviews and Meta‐analysis (PRISMA) Statement 2020 (Page et al. 2021) and the American Psychological Association (APA) reporting standards designed for MMSRs (Levitt et al. 2018).

## 3 Aim

To explore and compare clinicians' and patients' perceptions and experiences of the implementation of evidence‐based practice to improve clinical practice for preventing and managing SSIs within hospital acute care settings.

## 4 Methods

### 4.1 Search Methods

The search strategy was developed by two reviewers (CSW and EM) who have expertise in conducting MMSRs and an experienced senior librarian. The PICo (population, phenomenon of interest and the context) model guided the study selection eligibility criteria as it is a commonly used tool for constructing clinical research questions in association with evidence synthesis (Eriksen and Frandsen 2018). The PICo‐structured eligibility criteria are displayed in Table 1. The search term concepts related to (i) perceptions or experiences of preventing or managing, (ii) SSI and (iii) in acute hospitals were used in the search strategy.

**TABLE 1:** Study selection criteria based on the elements in the PICo questions.

	Population	Phenomena of interest	Context	Study type	Limits
Inclusion criteria	Hospital clinicians	Clinicians' views on preventing and managing SSI. Barriers and facilitators to evidence‐based practice for preventing and managing SSI.	Medical, surgical, general, subacute and rehabilitation wards.	Qualitative studies Quantitative studies Mixed‐methods studies	English language Empirical research Published since 2009
	Patients aged ≥ 18	Patients' perceptions and experiences of preventing and managing SSI.			
Exclusion criteria	Caregivers Student clinicians	Wounds or infections other than SSI. Studies only investigating the effectiveness of wound care products or devices.	Intensive care units, cardiac intensive care units, outpatient, palliative care, primary and community care.	Grey literature Conference abstracts Unpublished studies and dissertations	Not English Published prior to 2009

Abbreviation: SSI, surgical site infection.

Depending on the database, medical subject headings and keywords were used for each search term concept. The search term concepts were combined using Boolean ‘AND’, alternative spelling and synonyms were combined using Boolean ‘OR’. A comprehensive literature search was established for Medical Literature Analysis and Retrieval System Online (MEDLINE) and adjusted for the four other databases: EMBASE, Cumulative Index to Nursing and Allied Health Literature (CINAHL), PsycINFO and Cochrane Central Library. The search strategies for all databases are available in Data S1.

The initial literature search was undertaken on the 3 May 2021, with a literature search update performed on the 8 August 2022 and the final literature search update performed on the 23 March 2023. The references were imported into the MMSR software Covidence (*Veritas Health Innovation* 2021) to undertake screening, critical appraisal and data extraction.

Within MMSRs, to achieve maximum recall it is recommended to utilise a combination of databases (Bramer et al. 2017). Identification of relevant peer‐reviewed publications from conference and dissertation abstracts is necessary to ensure the inclusion of all relevant publications (Gentles et al. 2016). Conference and dissertation abstracts discovered via literature search, ProQuest Dissertations and Theses Global, Network Digital Library of Theses and Dissertations and Australasian Digital Theses through Trove and Open Grey databases were used to identify additional relevant peer‐reviewed publications. Bibliographies of the relevant MMSRs and included studies were searched to identify additional relevant references.

### 4.2 Inclusion and Exclusion Criteria

Original peer‐reviewed published empirical studies using any qualitative, quantitative or mixed methods research design were included if they (i) reported on hospital clinicians' or patients' perceptions and experiences of evidence‐based SSI prevention and management practices or barriers and facilitators to implementing SSI prevention and management guidelines or policies, (ii) were conducted in the setting of acute care hospital wards and (iii) were published in English peer‐reviewed journals since 2009. Grey literature, unpublished studies and dissertations were excluded from the MMSR. For this study, hospital clinicians are defined as health professionals who are directly involved in hospital patient care.

This review is part of a program of research underpinned by the Knowledge to Action Framework (Field et al. 2014) to (1) theoretically identify evidence‐practice gaps, (2) systematically assess barriers and facilitators to change at individual, organisational and system levels, followed by (3) select appropriate implementation strategies targeting the identified barriers and facilitators and (4) establish an intervention to enhance the adherence to SSI prevention and management guidelines in acute care hospital wards. Therefore, studies that were carried out in intensive care units, outpatient clinics, cardiac intensive care units, palliative care, primary and community care and patients aged under 17 or below were excluded. Studies that took place in the acute care hospital settings of medical, surgical, general, subacute and rehabilitation wards were included in this MMSR.

### 4.3 Quality Appraisal

The Mixed Methods Appraisal Tool (MMAT) was used to appraise the methodological quality of five types of study designs (qualitative research, randomised controlled trials, non‐randomised studies, quantitative descriptive studies and mixed‐methods studies) (Hong et al. 2018; Lizarondo et al. 2020). The quality improvement study was appraised using the validated Quality Improvement Minimum Quality Criteria Set (QI‐MQCS) (Hempel et al. 2015).

### 4.4 Data Extraction

The relevant data from all the included studies were extracted using a modified version of the Joanna Briggs Institute data extraction form, which was customised to this MMSR. Extracted data included: the first author's name, year of publication, study characteristics, participant characteristics and the qualitative and quantitative findings. From the qualitative studies, themes, findings on perceptions, experiences, barriers and facilitators and participant quotations were extracted. Data from open‐answer survey responses were extracted as qualitative data. From the quantitative studies, outcome measurement descriptions, result tables and summaries of narrative findings were extracted. The qualitative and quantitative data were extracted separately for mixed‐methods studies and quality improvement studies (Ackers et al. 2020).

Five reviewers (CSW, MM, HC, EH, AB) were involved in independently conducting the literature screening, data extraction and critical appraisal process. Two reviewers were randomly allocated for each study to be screened, data extracted and appraised. Any disagreements were discussed and outlying discrepancies were resolved by a reviewer who contained domain knowledge and methodological expertise (CSW).

### 4.5 Data Transformation

Following Stern et al. (2020) methodological guidance for MMSRs suggested by the Joanna Briggs Institute Handbook, a convergent integrated method incorporating data transformation of quantitative findings was utilised before data analysis. Qualitisation of quantitative data is regarded as suitable for transforming data into a collectively compatible format prior to data analysis to enable integration with qualitative data (Stern et al. 2020). The process of qualitisation can produce insights that can aid in understanding variations in the outcomes of research studies (Richards et al. 2019). One reviewer (EH) was responsible for the qualitisation of data. Original authors' narrative summaries of quantitative findings were used to guarantee the accuracy of the qualitised data.

### 4.6 Synthesis

This MMSR used a thematic synthesis approach to integrate and synthesise all the qualitised quantitative data and qualitative data (Thomas and Harden 2008). In accordance with the convergent integrated approach, the extracted qualitative and qualitised quantitative data carried equal weight (Thomas and Harden 2008). The qualitised data was gathered and analysed with the qualitative data from the original studies. Findings were initially line‐by‐line coded and then categorised by associated codes to define ‘descriptive’ themes (Thomas and Harden 2008). These ‘descriptive’ themes were shown as barriers and facilitators to implementing SSI prevention and management guidelines and were additionally aggregated and synthesised to produce overarching ‘analytical’ themes founded on ‘third order interpretation’ (Thomas and Harden 2008). These ‘analytical’ themes are the primary themes, with barriers and facilitators present under each key theme in the results section.

To begin, thematic synthesis was coordinated separately for the clinician and patient data by two reviewers (EH, AB) for all included studies. Any differences that emerged were discussed to reach consensus, with disagreements resolved by the reviewer with MMSR expertise (CSW). This was followed by comparative data analysis (Gibbs 2018) being commenced by comparing and distinguishing barriers and facilitators analysed and originated from clinicians' and patients' views under each primary theme. EH led the comparative analysis, with close conferral with the research team's involvement to provide investigator triangulation and attain consensus on findings.

## 5 Results

**FIGURE 1:**
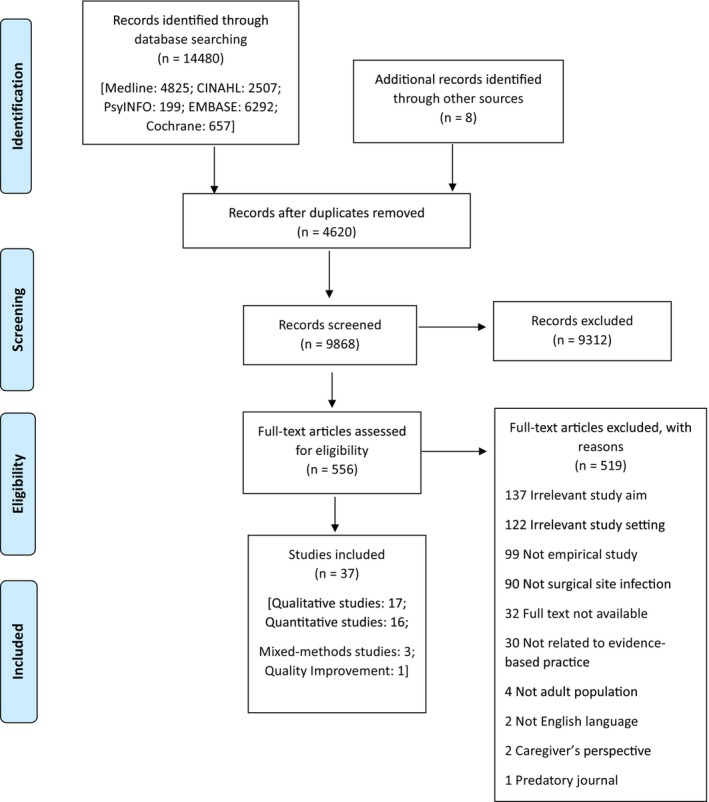
PRISMA flow diagram. [Colour figure can be viewed at wileyonlinelibrary.com]

The initial database search yielded 14,480 studies. Following duplicate removal and title and abstract screening, full‐text screening was undertaken on 556 studies (Figure 1). There were no additional relevant studies identified through hand searching from bibliographic data of included studies. A total of 37 studies were included.

### 5.1 Study Characteristics

Tables 2 and 3 show the study and participant characteristics of included studies (16 quantitative, 17 qualitative, 3 mixed‐methods, 1 quality improvement) containing hospital clinicians and patients. Except for one study that contained both clinicians' and patients' views on SSIs prevention and management (Walker et al. 2020), clinician and patient views were reported in separate studies. Studies were conducted in a range of countries, including Australia (*n* = 8), United Kingdom (*n* = 7), Canada (*n* = 3), Spain (*n* = 2) and United States (*n* = 2). The sample sizes ranged from 20 to 1005 participants in quantitative studies, 1 to 245 participants in qualitative studies, 13 to 207 participants in mixed‐methods studies and 25 participants in the quality improvement study (Ackers et al. 2020).

**TABLE 2:** Study and participant characteristics of studies that involved hospital clinicians, stratified by study design.

Author(s), year	Country	Methodology			Participants			Study focus
		Setting	Recruitment date	Data collection method(s)	Total number of participants	No. of participants in each health discipline	Years of practice (years)	
**Qualitative studies**								
Gagliardi et al. 2009	Canada	7 hospital sites	June to Nov 2007	Face‐to‐face interview (surgeons) Telephone interviews (managers)	19	7 Surgeons, 6 Infection managers, 6 Qualitative improvement managers	Not reported	Current SSI prevention practice to enhance quality improvement efforts
Gillespie et al. 2012	Australia	7 wards (4 surgical and 3 medical) in 1 hospital	Aug to Sep 2012	Environmental Scan: Macro approach	120	120 Ns	Median 10.5 (IQR 21) Range: 1–46	Knowledge and practices in the management of acute wounds
Charani et al. 2017	United Kingdom	30 WRs in 1 ICHNHS hospital	Not reported	Non‐participant observations Face‐to‐face interview	13	5 Consultant surgeons, 3 Registrars, 2 Ns, 2 Junior Dr., 1 Ward pharmacist.	Not reported	The impact of culture and team dynamics of the surgical ward rounds on antibiotic decision making
Kasatpibal et al. 2018	Thailand	25 government hospitals, 8 private hospitals	Nov 2013 to Feb 2015	Focus groups and face‐to‐face interviews	39 (Focus groups) 50 (Face‐to‐face interviews)	<Focus groups> 39 Theatre Ns <Interviews> 30 Ns (10 Theatre Ns, 10 Surgical ward Ns, 10 Ns Anaesthetists) 10 Surgeons, 10 Anesthesiologists,	<Focus groups> Mean 28.2 ± 4.1. <Interviews> Mean 24.7 ± 6.8	The experiences of surgical safety checklist implementation including barriers and possible facilitators
Lin et al. 2019	Australia	General surgical ward in 1 hospital	Dec 2014 to March 2015	Focus group and face‐to‐face interviews	20	20Ns (ENs & RNs in senior management roles)	Mean of 5 years Range: 1–14 years	The facilitators and barriers of adherence to evidence based wound care CPGs in preventing SSIs
Clack et al. 2019	Africa	5 hospitals	Not reported	Face‐to‐face interviews	13	5 Surgical team leaders, 3 Surgeon champions, 5 Ns champions	Not reported	The facilitators and barriers of implementing SUSP and its effects on cultural safety
Ierano et al. 2019	Australia	1 private hospital, 2 public hospitals	Not reported	Focus groups and face‐to‐face interviews	77	13 Theatre Ns, 10 Anaesthetists, 40 Surgeons, 8 Pharmacists, 6 Surgical registrars and residents	Not reported	The barriers and enablers of appropriate SAP prescribing and evidence‐based guideline compliance
Troughton et al. 2019	United Kingdom	3 hospitals belonging to the ICHNT hospital group	May 2017 to July 2018	Face‐to‐face interviews	20	1 Anaesthetist, 1 Microbiologist, 6 Ns, 1 Pharmacist, 8 Surgeon, 3 Theatre personnel	Range: 3–31 years	Health care professional views of SSI prevention including barriers and facilitators and the determinants of IC behaviours including contextual, environmental and social factors
Vieirade Souza and Serrano 2020	Brazil	1 general surgery service unit in 1 hospital	Dec 2018 to Jan 2019	Face‐to‐face interviews	9	9 Ns	Mean workload of 42.22 h per week. Range: 1–29 years	The experiences of preventing SSI in nursing
Walker et al. 2020	Australia	1 hospital	Not reported	Modified NGT through workshops	4	1 Dr., 3 Ns	Not reported	The priorities and challenges related to surgical wound care
Mmari et al. 2021	Tanzania	1 hospital	Not reported	Face‐to‐face interviews	14	Surgical resident: 2; Obstetrics and gynaecologist: 3; General surgeon: 2; Urologist: 2; Otolaryngologist: 2; Neurosurgeon: 1; Ophthalmologist: 1; Orthopaedic surgeon: 1	< 2 years: 3 2–10 years: 11	To describe the perceptions of Surgeons on surgical antibiotic prophylaxis use at an urban tertiary hospital.
**Quantitative studies**								
Eskicioglu et al. 2012	Canada	5 academic teaching hospitals, 2 community‐affiliated hospitals	Not reported	Developed survey	76	40 Surgeons 36 Surgical residents	<Surgical residents> 1st year: 21% 2nd: 71% 3 nd:89% 4th: 44% 5th: 50%	The awareness, agreement, adoption of and adherence to SSI prevention strategies
Gillespie et al. 2014	Australia	1 hospital, 7 wards (4 surgical and 3 medical wards)	Aug 2012 to Sep 2012	42‐item survey based on an extensive literature review and an environmental scan of wound care issues in major hospitals	120	120 Ns	Median 10.5 years (IQR 21 years), range: 1–46 years	To describe Ns self‐reported knowledge and practices in the management of acute wounds
Pucher et al. 2014	United Kingdom	1 hospital	Not reported	Survey with intervention group	20	20 General surgical registrars (Registrar‐level trainees)	Length of surgical training (years) Control: Median 3 (2–4) Intervention: 3 (2–4)	To evaluate a checklist‐based tool to improve and standardise care of postoperative complications
Accardi et al. 2017	Italy	16 medical and surgical wards in 1 hospital	Dec 2015 to Feb 2016	Questionnaire and Survey	128	128 Ns	<Work experience in current ward> < 1: 14.8% 1–5: 27.3% 6–10: 22.7% > 10: 35.2%	The knowledge level of healthcare‐associated infection and compliance of the evidence‐based prevention
Ding et al. 2017	Australia	1 hospital	Feb to April 2014	Observation, reviewing the medical records	60	60 Surgical Ns	Not reported	To prospectively describe surgical Ns postoperative wound care practices and the extent to which observed surgical wound practices guideline aligned with evidence‐based recommendations
Balodimou et al. 2018	Greece	Surgical departments in 1 hospital	May to August 2016	Validated anonymous, self‐completion questionnaire	148	148 Ns	0–5: 21% 6–10: 17% 11–15: 21% 16–20: 16% 21–25: 12% 26–30: 14%	Nurses' knowledge of SSI prevention
Moran and Byrne 2018	Ireland	Cardiothoracic surgical units in 8 hospitals	Not reported	Validated questionnaire	158	158 Ns	<Years of RN experience in Public/Private hospital> ≤ 5: 14.6%/ 17.6% 6–10: 15.4%/ 26.9% 11–15: 26%/23.5% 16–20: 22%/20.6% > 20: 22%/11.8%	Current knowledge of wound care in post cardiac surgery
Ahmed et al. 2019	Sudan	Surgery department at 1 hospital	Dec 2017 to Jan 2018	Self‐administered four section, multiple‐choice questionnaire	49	39 Surgical registrars 10 Anesthesiologists	Mean 2 ± 1.5	The knowledge, attitude and adherence to the practice of SAP guidelines
Badia et al. 2020	Spain	Not reported	Jan 8 to Feb 28, 2018	Web‐based survey questionnaire	355	355 Colorectal Surgeons	< 10 year: 165/351 (47%); > 11 year: 255/351 (53%)	To evaluate the knowledge, opinions and practices of colorectal surgeons on preventive measures including mechanical bowel preparation, oral antibiotic prophylaxis and the use of drains.
Ryu, Yoo, and Choi 2020	Korea	Not reported	July to August 2018	Developed survey's	16 (1st survey) 15 (2nd survey)	16 Surgeons (4 Orthopaedics, 3 General surgery, 2 Infectious disease, 2 Cardiothoracic Surgery, 5 Others)	< 1st survey> Mean 21.13 ± 7.45 < 2nd survey> 20.67 ± 7.47	Physicians' perceptions of SSI assessment indicators and the barriers of implementation
Badia et al. 2020	Spain	Not reported	Not reported (the web‐based survey was open for 60 days)	Self‐developed web‐based survey questionnaire	1105	Operative Ns (25.4%), Surgeons: Vascular (6.9%), Cardio‐thoracic (9.2%), Oncology (6.3%), Plastic/ Aesthetic (6.7%), Coloproctology (9.7%), head and neck (7.6%), paediatric (2.8%), neurosurgery (13.8%), obesity (10.7%), general (27.9%) 11 associations of Operative Ns and Surgeons	< 20 years: 687/1087 (63.2%) > 20 years: 400/1087 (36.8%)	To understand the current level of compliance with these guidelines by a range of surgical specialists, prior to grouping the most important preventative measures into bundles and planning a dissemination strategy that could increase their level of implementation at a national level.
Ghuman et al. 2021	Canada	Not reported	Not reported	Online 23 question survey based on WHO SSI guidelines, input from colorectal Surgeons and a Harvard University Survey Research Methods expert	97	97 BC Surgeons	Not reported	To document whether SSIs are being monitored, identify current SSI rates and determine what strategies BC surgeons use for SSI prevention in colorectal surgery.
Altaweli et al. 2023	Saudia Arabia	4 hospitals	Jan to May 2020	Developed a structured questionnaire	360	360 Ns	< 6 years: 110 (49.80%) > 6 years: 111 (50.23%)	The overarching objective of this study was to assess the knowledge and practice of nursing staff regarding various aspects of the management of acute surgical wounds
**Mixed‐methods studies**								
Sickder et al. 2017	Bangladesh	General and orthopaedic departments in 3 hospitals	June to Oct 2015	Developed validated questionnaire and survey. Focus group and face‐to‐face interviews	182 (Survey) 22(Focus group) 3 (Interview)	182 RNs (Survey) 22 Ns (Focus group) 3 Ns administrators (Interviews)	Not reported	Current practices for SSI prevention including barriers and facilitators and the directions of improvement
Lin et al. 2019	Australia	1 surgical ward in 1 hospital	2015 to 2016.	Focus groups and individual face‐to‐face interviews	19 (17 Focus group, 2 Interview)	17 Ns (RNs and ENs) 2 Senior Ns	Mean 4.7 ± 3.7	The effectiveness of implementing multi‐component intervention designed to prevent SSI
Do, Edwards, and Finlayson 2021	Vietnam	4 surgical wards in 1 hospital	1st Feb to 30th April 2016	Retrospective clinical chart audit and semi‐structured interviews	13	13 Surgical Ns	Median 9 (2–35) Surgical experience (years): Median 8 (2–35)	To explore acute care Ns perceptions on factors constraining adequate wound assessment documentation
**Quality improvement studies**								
Ackers et al. 2020	Uganda	Postnatal and gynaecology ward in 1 hospital	Jan 2019 to Jan 2020	Face‐to‐face interviews and observation (action‐research intervention using multi‐method ethnography)	25	50% of the Ns, Midwives, Intern Dr., Laboratory technicians and Pharmacists, 2 hospital managers and 3 UK volunteers	Not reported	The improvement strategy for antimicrobial stewardship

Abbreviations: BC, British Columbia; CABGs, coronary artery bypass surgery; CAUTI, catheter‐associated urinary tract infection; CLABSI, central line‐associated bloodstream infection; CPGs, Clinical Practice Guidelines; ENs, enrolled nurses; IC, infection control; ICHNHS, Imperial College Healthcare National Health Service; ICHNT, Imperial College Healthcare National Health Service Trust; NGT, nominal group technique; Ns, Nurses; OR, operating room; RNs, registered nurses; SAP, surgical antibiotic prophylaxis; SAP, surgical antimicrobial prophylaxis; SSIs, surgical site infections; SUSP, Surgical Unit‐Based Safety Programme; WHO, World Health Organisation; WRs, ward rounds.

**TABLE 3:** Study and participant characteristics of studies that involved patients, stratified by study design.

Author(s), year	Country	Methodology				Participants			Areas of interest
		Setting	Recruitment date	Data collection method(s)	Total number of patients	Age, year	SSI risk/wound status	SSI prevention/treatment strategies	
**Qualitative studies**									
Mottram 2011	United Kingdom	2 day‐surgery units	2004 to 2006	Two semi‐structured interviews which took place by telephone 48 h and 1 month, respectively, following surgery	145 pts.' and 100 carers	Not reported	Not reported	Wound care after undergoing day surgery	To explore pts.' experiences following discharge from the day surgery unit
Tanner et al. 2012	United Kingdom	3 acute hospitals	Not reported	Unstructured interviews	17	Not reported	<Wound status> 1 superficial; 16 deep or organ space	Not reported	The experience of pts.' with an SSI.
Tanner et al. 2013	United Kingdom	3 hospitals	2011 to 2012 (Over 5 months)	Narrative‐based face‐to‐face interviews	17	30–39: 2 40–49: 2 50–59: 2 60–69: 8 70–79: 1 80–89: 2	< SSI classification > 10 Organ space 2 Uterine 1 Superficial 4 Deep	Not reported	Pt experiences of SSIs for improving clinical practice.
Brown, Tanner, and Padley 2014	United Kingdom	1 hospital	Not reported	Semi‐structured interviews face‐to‐face	17	31–40: 2 41–50: 2 51–60: 2 61–70: 8 71–89: 1 81–90: 2 (Unclear if overlap exists in the last 2 categories)	Not reported	Not reported	The pt. experience of suffering from an SSI.
Gelhorn et al. 2018	United States	1 single clinical site	Not reported	A focus group and one‐on‐one telephone interviews	15 pt.'s in a (*n* = 3) focus group and 12 individual telephone interviews	Mean (SD): 56.7 (10.6)	Spinal (*n* = 4), knee (*n* = 3) or hip (*n* = 8) Replacement surgery participated in one focus group (*n* = 3) and 12 individual telephone interviews.	Not reported	To understand the pt. experience by gathering qualitative data to characterise the relevant burden of illness issues and impacts among spinal, knee replacement or hip replacement surgery pts.' who have experienced SSIs
Walker et al. 2020	Australia	1 hospital	Not reported	Face‐to‐face interview	1	Not reported	Receiving care for a wound (Does not reported about the wound condition)	Not reported	The priorities and challenges related to surgical wound care between perspectives of clinicians and health consumers/pts
Larsson, Nyman, and Brynhildsen 2023	Sweden	Department of vascular/cardiothoracic surgery in 1 hospital	May to Dec 2018	Face‐to‐face interviews	16 (13 men, 3 women)	49–85 years (mean 68.5)	Not reported	Pt experiences of SSIs	To describe pt. experiences associated with acquiring a severe infection in the harvesting site after CABG
**Quantitative studies**									
Merle et al. 2011	France	1 surgical ward in 1 hospital	Oct to Nov 2005 and Apr to Aug 2006	Information leaflet and interviews	161 (87‐IG, 74‐CG)	IG: 52.3 ± 16.6 CG: 56 ± 14.9	< Occurrence of surgical wound infection > IG: *n* = 13(15%) CG: *n* = 3(4%)	IG: Receiving oral information of SSI and leaflet during the preoperative visit. CG: Receiving oral information only.	The effectiveness of written information about SSI in pts.' satisfaction and recollection in addition to oral information.
Anderson et al. 2013	United States	1 acute care hospital	July to Oct 2011	Developed survey	50	≦ 64: 84%	Previous SSI: *n* = 5 Previous healthcare‐associated infection other than SSI: 8%	Not reported	Pts' awareness and knowledge regarding risks and consequences of and prevention of SSI.
Cooper et al. 2019	Australia	Adult surgical inpatients in 1 hospital	Sep to Oct 2017 (5‐weeks)	Developed cross‐sectional survey, patient information sheet and a standardised script to guide preadmission	222	Range: 18–97	Not reported	In preadmission, receiving an information sheet on CHG washes by email or in person. In follow‐up, phone call and email templates.	Improving pt. understanding of and patient compliance with evidence‐based guidelines for CHG preoperative washes.

Abbreviations: CABG, coronary artery bypass surgery; CG, control group; CHG, chlorhexidine gluconate; IG, intervention group; *n*, number; Ns, nurses; Pt, patient; SSI (s), surgical site infection(s).

Of the 28 studies with clinicians' views, 12 quantitative studies used validated SSI knowledge and/or attitude questionnaires or self‐developed surveys to investigate the knowledge, attitude and/or barriers to SSI prevention. Of those, only two studies also investigated SSI management (Gillespie et al. 2014; Altaweli et al. 2023). Of the 11 clinician‐related qualitative studies, 7 studies used face‐to‐face interviews or focus groups, one study used face‐to‐face‐interviews and telephone interviews (Gagliardi et al. 2009), one study face‐to‐face‐interviews and non‐participant observations (Charani et al. 2017), one study used an environmental scan approach (Gillespie et al. 2012) and one study used a modified nominal group technique through workshops (Walker et al. 2020) to explore views on SSI prevention. One qualitative study also explored SSI management (Gillespie et al. 2014). The three clinician‐related mixed‐methods studies used developed validated questionnaires, surveys, focus groups, face‐to‐face interviews, semi‐structured interviews and retrospective clinical chart audit to investigate practices and views on SSI prevention and management plans (Sickder et al. 2017, Lin et al. 2019, Do, Edwards, and Finlayson 2021). The one quality improvement study (Ackers et al. 2020) used face‐to‐face interviews and observational work to explore the improvement strategy for antimicrobial stewardship.

Of the 10 studies with patients' perspectives, seven were qualitative studies that used semi‐structured interviews which took place by telephone, narrative, unstructured or structured face‐to‐face interviews, or a focus group that explored their views on SSI prevention received while in hospital. No qualitative study reported patient views on SSI management. The remaining three quantitative studies used cross‐sectional surveys, information leaflets, a standardised script to guide preadmission and/or interviews to investigate patient awareness or improve patient understanding and compliance with evidence‐based SSI clinical guidelines (Merle et al. 2011; Anderson et al. 2013; Cooper et al. 2019).

### 5.2 Participant Characteristics

Data from 3369 clinicians and 673 patients were reported. Of the 28 studies that recruited clinicians, 12 recruited only nurses; nine recruited various clinicians, including nurses and physicians and/or allied health and seven recruited only physicians. Nineteen studies reported years of clinical practice, ranging from less than 1 year (Accardi et al. 2017; Balodimou et al. 2018) to 46 years (Gillespie et al. 2012, 2014). It was possible to compare views between health professionals, as the participants' health disciplines were clearly reported in the participant demographics. It was not possible to compare views between health professionals in relation to the quality improvement study (Ackers et al. 2020) as some participants' health disciplines were not clearly reported. Of the 10 patient‐related studies, seven reported participants' ages ranging from 18 to 97 (Cooper et al. 2019).

Six studies reported on the patients' experience with an SSI (Mottram 2011, Tanner et al. 2012, Tanner et al. 2013, Brown, Tanner, and Padley 2014, Gelhorn et al. 2018, Larsson, Nyman, and Brynhildsen 2023). Two studies explored patients' awareness and knowledge of SSIs and patient compliance with evidence‐based guidelines (Anderson et al. 2013, Cooper et al. 2019). One study discussed patients' satisfaction regarding written information or oral information about SSIs (Merle et al. 2011). One study presented the priorities and challenges associated with SSI care for both patients' and clinicians' (Walker et al. 2020).

### 5.3 Methodological Quality of Included Studies

Quality assessment results using MMAT are presented in detail in Data S2. Overall, of the 17 included qualitative studies, the majority displayed consistency within each study, between the study aim, data collection, analysis and interpretation and presented information on data analysis and interpretation. Among the qualitative studies, seven studies provided no or unclear details of data collection methods to validate how research questions were addressed, four studies included clinician views (Gagliardi et al. 2009; Gillespie et al. 2012; Troughton et al. 2019; Vieirade Souza and Serrano 2020), three studies consisted of patient views (Brown, Tanner, and Padley 2014; Gelhorn et al. 2018; Tanner et al. 2012).

Of the 16 included quantitative studies, most studies illustrated use of relevant measurement tools and statistical analysis to answer the research question. Six studies had a low risk of nonresponse bias, three comprised patient perspectives (Anderson et al. 2013; Cooper et al. 2019; Merle et al. 2011) and three consisted of clinician views (Ahmed et al. 2019; Ghuman et al. 2021; Pucher et al. 2014). Two quantitative studies which comprised clinician perspectives demonstrated that the study sample was representative of the target population (Accardi et al. 2017; Altaweli et al. 2023).

None of the three mixed‐methods studies (Do, Edwards, and Finlayson 2021, Lin et al. 2019, Sickder et al. 2017) which all included clinician views distinctly reported how the combination of qualitative and quantitative findings was achieved.

The quality improvement study (Ackers et al. 2020) which consisted of clinician perceptions met the standard of 12 domains out of the 16 QI‐MCQS domains and provided no or little information about the study design, patient health‐related outcome measurement, the study methods are not described in sufficient detail to be replicated and study design limitations (see Data S3).

### 5.4 Themes

Five main themes were created from the clinicians' and patients' views. In each theme, clinicians' views are concurrent with patients' views. The five themes are: (1) Intentional non‐adherence to insufficiently detailed and outdated guidelines, (2) knowledge deficits on evidence‐based SSI care bring about inconsistent clinical practice, (3) collaborative interdisciplinary and patient‐provider relationship to enhance guideline uptake, (4) infection surveillance to improve patient safety and quality of life and (5) negative physical and psychological impacts on patients.

#### 5.4.1 Theme 1: Intentional Non‐adherence to Insufficiently Detailed and Outdated Guidelines

SSI evidence‐based prevention and management guidelines are viewed as vital by physicians and nurses. However, these healthcare professionals express concerns regarding hospital clinical guidelines as they are often not up‐to‐date and insufficiently detailed guidelines to follow and therefore can lead to patient dissatisfaction around wound care delivery. Awareness around SSI prevention and management guidelines remains a growing issue as numerous physicians and nurses are unaware of what specific guidelines to follow. This is showcased in studies describing clinicians' views as they reveal that SSI prevention and management guidelines are readily available however they are not followed or adhered to (Moran and Byrne 2018), or not known about (Gillespie et al. 2013).

For example, a study conducted by Altaweli et al. (2023) revealed that less than two‐thirds of nurses always followed national or international guidelines associated with wound care management. The practice aspect of ‘the nurses’ in the Altaweli et al. (2023) study was poorer than the knowledge aspect, revealing a transparent gap is evident between the proposed guidelines and real‐life practices of nursing staff. Examples of the nature of non‐adherence to SSI prevention and management guidelines occurred in the context of dressing removal post‐surgery as the guideline contained knowledge gaps, not specifying when the dressing is to be removed (Moran and Byrne 2018), and the timing of prophylactic antibiotics due to physicians lack of knowledge and guideline adherence (Ahmed et al. 2019).

In some instances, guideline non‐adherence resulted from lack of guideline knowledge (Gillespie et al. 2013), the unavailability of local guidelines (Mmari et al. 2021) and the absence or non‐existence of national guidelines (Sickder et al. 2017).

‘No, I have never seen guidelines…’. (Mmari et al. 2021, 6).

Other studies identified non‐adherence to guidelines as intentional. Intentional non‐adherence emerged for a variety of reasons including guidelines being outdated and lacking sufficient detail (Lin et al. 2019), insufficiently detailed guidelines that accounted for the complexity of individual patient circumstances (Ierano et al. 2019), high levels of discrepancy between published guidelines and actual clinical practice (Badia et al. 2020) and the perception of gaps between the guidelines and current evidence (Ierano et al. 2019).

‘Even though there might be recognised guidelines, many … have their own preferences…they will override’. (Ierano et al. 2019, 6)

Guiding principles were viewed as important in wound care (Walker et al. 2020), and using a standardised approach aligned to evidence was shown to reduce error (Pucher et al. 2014). Guidelines for SSI prevention and management, when accessible were a facilitator for junior health care physicians (Ierano et al. 2019). Therefore, compliance with guidelines needs to improve to ensure adequate prevention and management of SSIs (Badia et al. 2020 ^a^).

#### 5.4.2 Theme 2: Knowledge Deficits on Evidence‐Based SSI Care Bring About Inconsistent Clinical Practice

Inadequate evidence‐based knowledge about SSI prevention and management led to inconsistent clinical practice in wound care among physicians and nurses. This knowledge deficit encompassed most aspects of wound care including prevention of SSI, wound assessment, wound management, the use of prophylactic antibiotics, documentation requirements and the use of primary evidence. Patients were aware of the inconsistent wound care practices among physicians and nurses and expressed a lack of SSI knowledge themselves.

Ten physician and nurse‐related studies reported on the management of surgical wounds, describing the existence of knowledge deficits (Gillespie et al. 2012, Sickder et al. 2017, Moran and Byrne 2018, Vieirade Souza and Serrano 2020, Altaweli et al. 2023) and the inconsistent use of guidelines in the health profession especially between physicians and nurses (Gillespie et al. 2013; Pucher et al. 2014). Ahmed et al. (2019) study explored both the existence of knowledge deficits and inconsistent use of guidelines between physicians, the poor use of documentation (Do, Edwards, and Finlayson 2021) and the importance of interdisciplinary wound care teams (Walker et al. 2020). Addressing knowledge deficits is crucial, and meaningful collaboration between physicians and nurses is an important facilitator in closing gaps in knowledge (Walker et al. 2020).

‘I believe that knowledge is the greatest facility, knowing my work and the procedures’. (Vieirade Souza and Serrano 2020, 13)

The knowledge of wound management was described generally as either a practice gap in nurses' knowledge of acute wound care (Gillespie et al. 2013), a significant knowledge deficit in wound management (Moran and Byrne 2018) or insufficient knowledge regarding SSI prevention (Sickder et al. 2017). The practice of wound assessment was often poor, with limitations on assessment of wound physiology as well as clinical signs of infection identified (Altaweli et al. 2023). In one study, this knowledge deficit about wound assessment was seen to emerge from a lack of continuing education (Do, Edwards, and Finlayson 2021). Physicians in the Ahmed et al. (2019) study were found to have knowledge deficiencies about appropriate surgical antibiotic prophylaxis, particularly related to the first‐line choice antibiotics, these knowledge deficiencies were attributed to inconsistencies with the available scientific evidence as majority of the physicians utilised textbooks or articles as their primary source of knowledge.

This knowledge deficit was also evidenced within the context of nursing documentation with Do, Edwards, and Finlayson (2021) identifying the poor ability to describe wound assessment and documentation about wound care, with nurses reporting it as ‘unimportant’. In two studies, the organisations wound care specialist was identified as the most important source of information regarding wound care (Gillespie et al. 2013; Moran and Byrne 2018). Gillespie et al. (2013) report 75% of respondents used wound care specialists as their primary source of information. Fewer than half of the nurses in Altaweli et al. (2023) study did not consider resources such as policies or journal articles to support evidence‐based practice, preferring peer knowledge support from wound care specialists.

Education strategies such as posters and scenario‐based quizzes were identified as facilitators for nurses in improving aseptic techniques in wound care practices (Lin et al. 2019). Participants in Lin et al. (2019) identified educational resources beneficial to improving their skills with aseptic non‐touch techniques. Conversely, a lack of education was identified as a barrier:

‘I never attend any educational workshops related to wound assessment and management in the hospital. Therefore, nurses are lacking knowledge in wound assessment’. (Do, Edwards, and Finlayson 2021)

Some challenges with documentation were tied to knowledge deficits:

‘… a lot of big words in the Wound Care Pathway, and some of them I don't even know what they mean’. (Lin et al. 2019)

Insufficient patient knowledge about SSI prevention and management was also identified. In some instances, patients lacked overall knowledge about the different types and treatments of SSIs, and patients raised the need for overall awareness, knowledge and understanding of SSIs as it could lead to greater compliance with preventative and management interventions from their perspectives (Tanner et al. 2013; Gelhorn et al. 2018):

‘I was aware that there was a possibility of infection with any surgery. As far as staph what that means, no, that was a Google adventure that happened’. (Gelhorn et al. 2018, 359)

‘I was on an antibiotic drip but I just assumed that was normal after an operation’. (Tanner et al. 2013, 43)

In one study, 16% of surgical patients identified no recollection of education about SSIs, and 26% thought patient education regarding SSI prevention required improvement (Anderson et al. 2013, 1294). However, 94% of patients in the same study felt comfortable with their knowledge of SSIs risks and consequences (Anderson et al. 2013, 1294).

Some patients expressed satisfaction with the degree of resources shared for instance photographs of their wound assessment and progression:

‘They kept me informed of progress and things like that. When they started, the hole was five centimetres deep and it's now gone’. (Walker et al. 2020, 19)

‘…so that I could…follow the progress, so I was fully informed’. (Walker et al. 2020, 19)

Surgical wound care prevention and management education provided to patients in preparation for surgical intervention was reported in two studies. Merle et al. (2011) examined the effectiveness of written information about SSIs and identified that patient knowledge about SSIs was improved following the provision of written information—however, patients with a poor understanding of the concept of hospital infection experienced fewer improvements. Similarly, Cooper et al. (2019) found pre‐operative education generally beneficial however, it was limited by the provision of inconsistent information delivery.

#### 5.4.3 Theme 3: Collaborative Interdisciplinary and Patient–Provider Relationship to Enhance Guideline Uptake

Collaborative interdisciplinary SSI care and desirable patient‐provider relationship were facilitators to improve evidence‐based SSI care according to physicians, nurses, allied health professionals and patients' perspectives. Clinicians expressed that multidisciplinary team function and the effectiveness of communication between clinicians and patients as integral parts of SSI prevention and management. Crucial factors here included the accessibility of wound care specialists, interdisciplinary team education and knowledge about SSI prevention and management, skills to build and maintain desirable patient‐provider relationships and interdisciplinary team collaboration supported effective SSI wound management practices (Sickder et al. 2017).

A key element of effective collaborative practices is communication within the team. Inadequate team support, non‐comprehensive documentation and insufficient time were reported as a challenge to effective communication. Open dialogue through verbal communication and the exchange of experiences accentuated physicians, nurses and allied health professionals' capacity for empathy through listening and being present (Mosse et al. 2023), thereby improving health professionals' knowledge and clinical practices (Vieirade Souza and Serrano 2020).

However, within the context of prescribing prophylactic antibiotics, the professional roles of healthcare professionals were seen as challenging in this collaborative approach. Mmari et al. (2021) identified differences of opinion between healthcare professionals' roles within the decision‐making process. Some healthcare professionals specified that decision‐making should be a team approach:

‘I think this has to be team work, from the pharmacist, the resident…’. (p.8)

Others expressed that the decision‐making role should be designated to one specific clinician, the surgeon alone, as the prescriber to be responsible:

‘So, the surgeon should decide’. (p.7).

For nurses, documentation was an essential communication tool and pivotal in monitoring SSIs (Ding et al. 2017) and implementing wound care management practices (Do, Edwards, and Finlayson 2021). However, there were ‘disagreements on where and what to document…’ on standardised wound care templates, based on the nature of the wound that existed, due to nurses in the study being unclear about what and where to document certain wounds (Lin et al. 2019). Other barriers to documentation use included: retrospective and incomplete documentation (Ding et al. 2017), inconsistency in what was required to be documented, perceived unimportance of documentation (Do, Edwards, and Finlayson 2021) and time shortages (Lin et al. 2019).

From the patients' perspective, a lack of collaborative clinical support beyond the acute care setting to help transition from hospital to home was identified as a barrier to maintaining desirable patient‐provider relationships (Mottram 2011; Tanner et al. 2012).

‘If someone came round and said to you as you were discharged right here are some numbers, a list of all the things you could go through if there are any problems, but they just discharge you … it's frightening when you come home and there is nobody’. (Tanner et al. 2012, 167)

Of particular importance to patients was the continuous information sharing between clinicians and patients throughout the hospital stay in providing high‐quality patient care.

‘…being kept informed and the caring and sharing attitude of the people involved…’. (Walker et al. 2020, 19)

For some, clinicians' reluctance to engender forthrightness within the relationship was seen as a negative experience.

‘I wasn't told I had an infection…’, and ‘…don't like admitting to infections’. (Tanner et al. 2013, 43)

#### 5.4.4 Theme 4: Infection Surveillance to Improve Patient Safety and Quality of Life

Infection surveillance in hospitals was important to monitor, facilitate the implementation of evidence‐based SSI practices, and subsequently improve patient safety and quality of life. They were viewed as essential by patients who had experienced SSIs previously.

Gillespie et al. (2012) conducted an environmental scan exploring drivers and barriers to surgical wound management, with the primary drivers identified as infection surveillance, interdisciplinary collaboration, regulatory mechanisms and product choice. Specifically, infection surveillance was identified as important for identifying SSI occurrence, identifying causal links, comparing interventions and guiding infection control practices (Gillespie et al. 2012). However, over half of surgeons in Badia et al. (2020) qualitative study identified not receiving feedback on SSI rates. A lack of SSI feedback systems (Sickder et al. 2017) and inadequate data to support practice (Mmari et al. 2021) were also identified as a barrier to effective SSI management. The benefits of receiving feedback on hospital practices are recognised by healthcare professionals in Troughton et al. (2019) study exploring determinants of infection control practices:

‘…suddenly the problem is made visible…[before] no one notices or people pretended not to notice…’. (p.6)

Infection surveillance is important as it can help develop a patient safety culture, work prioritisation in relation to SSI prevention and identify hospital settings that are short staffed early. This is vital as lack of time hindered patient wound care education (Brown, Tanner, and Padley 2014; Gelhorn et al. 2018) and insufficient time related to patient load, nursing shortage and excessive administrative tasks reduced the capacity to document wound care appropriately (Do, Edwards, and Finlayson 2021, 8).

#### 5.4.5 Theme 5: Negative Physical and Psychological Impacts on Patients

Six studies reported on the patients' experience with an SSI (Mottram 2011, Tanner et al. 2012, Tanner et al. 2013, Brown, Tanner, and Padley 2014, Gelhorn et al. 2018, Larsson, Nyman, and Brynhildsen 2023). A diverse range of impacts from SSI experiences were reported by patients, both physical and psychological. Physical impacts included pain and malodour:

‘…you are in agony’. (Tanner et al. 2012, 165)

‘It was really stinking and I couldn't look at it’. (Tanner et al. 2012, 165)

The psychological impacts of navigating the experience of an SSI were prevalent, ranging from noticeable to very significant. These included a sense of helplessness; impact on family members; social isolation and unexpected financial costs. Within the context of day surgery, and reporting on the occurrence of an SSI after discharge, a participant in Mottram's (2011) study stated:

‘I didn't know what to do…if only a nurse could have had a look…’. (p.3147)

The psychological effects were more evident for some:

‘I felt a little depressed…’. (Larsson, Nyman, and Brynhildsen 2023, 15)

‘I felt really down, utter despair’. (Tanner et al. 2012, 166)

Two studies reported the psychological effect as being seen to be potentially positive, dependent upon the outlook of the patient:

‘a sense that the SSI occurred through chance or happenstance equated to a stoic outlook’. (Brown, Tanner, and Padley 2014)

‘The fact that I have got rid of my cancer you almost forget about [the infection]’. (Tanner et al. 2012, 166)

Understanding the diverse attitudes patients have towards SSIs is vital as awareness, understanding and concern about SSIs have been identified as lacking (Tanner et al. 2013). Researching patient‐specific attitudes concerning SSIs raises awareness among patients and healthcare clinicians, which can be utilised within healthcare to enhance SSI prevention and management practices (Mottram 2011).

## 6 Discussion

This MMSR synthesises and collates clinicians' and patients' experiences and views on implementing SSI prevention and management guidelines, providing insights on aspects that influence the uptake of evidence‐based SSI prevention and management from the point of view of clinicians and patients in acute care settings. The findings of this review imply the significance of a multifaceted intervention that addresses barriers to improve (1) patient‐provider interaction, (2) clinicians' knowledge and accessibility to updated guidelines, (3) interdisciplinary collaboration and (4) infection surveillance in SSI prevention and management.

Optimal patient‐provider interaction is highlighted in this review as healthcare professionals directly influence patients' SSI experiences by educating and encouraging them to be involved in SSI prevention and management shared decision‐making (Tartari et al. 2017). Patient‐provider interaction and patient understanding of SSI influence patients' preferences for the prevention and management of SSIs (Rawson et al. 2016). Understanding patient preferences is expected to promote patient contentment and patient‐centred care (El‐Haddad, Hegazi, and Hu 2020) and improve guideline implementation in a patient‐centred manner (Oben 2020). Hence, healthcare professionals are accountable for advancing and refining their communication skills, being actively engaged, available for patients' queries to aid in an optimal patient experience (Grocott and McSherry 2018). Failing to include and engage patients in the decision‐making process about their SSI prevention and management can lead to misinformation, frustration, anxiety and non‐adherence to SSI guidelines (Rawson et al. 2016). Therefore, healthcare professionals need to consider patients' cultural and healthcare preferences, and health literacy needs to ensure the information they deliver is culturally appropriate and tailored to individual needs (Tartari et al. 2017).

In addition, this review shows that non‐adherence to SSI prevention and management guidelines can be inadvertent or intentional by clinicians due to insufficiently detailed and outdated guidelines for preventing and managing SSIs in acute care hospital settings. The successful uptake of Clinical Practice Guidelines (CPGs) is influenced by clinicians' entrusting the quality and integrity of the content displayed in an accessible and practical manner (Gillespie et al. 2018). Clinicians, specifically surgeons, commonly do not adhere to SSI guidelines due to guideline inflexibility restricting autonomous practice associated with reflection and self‐awareness to fulfil guidelines and checklists (Leaper et al. 2014), as well as the lack of evidence‐based policies and procedures, resource‐ and funding‐limited, poor patient and clinician awareness of their responsibilities (Morikane et al. 2021). The impact of SSI CPGs on improving patient outcomes depends on the extent to which clinicians follow these guidelines (Gillespie et al. 2018; Tomsic et al. 2020). All hospitals should continuously and proactively work towards improving guidelines adherence by entrenching effective, relevant and feasible SSI prevention and management strategies within their organisational context (Morikane et al. 2021).

Concurring with our findings, clinicians acknowledging their professional role in guideline implementation is fundamental in improving interdisciplinary collaboration in guideline uptake (Hahn and Truman 2015; Horgan, Saab, et al. 2023). The updated knowledge across health disciplines regarding SSI prevention and management guidelines should be shared to ensure best practice is delivered (Sartelli et al. 2020). Healthcare professionals are responsible for continuing their education to ensure they are up to date with knowledge and skills (Mlambo, Silén, and McGrath 2021). Clinical champions or opinion leaders who have updated SSI knowledge may potentially improve evidence‐based SSI prevention and management, which requires further research to evaluate its effectiveness (Horgan, Saab, et al. 2023).

Furthermore, this review highlights the importance of collaborative interdisciplinary SSI care to improve evidence‐based SSI prevention and management care. Like other areas of clinical practice, the way the multidisciplinary team functions and communicates is vital in improving patient safety and reducing SSIs (Dellinger 2016). The use of SSI intervention bundles targeting three to five small achievable clinical goals and implementation strategies can assist clinicians, specifically nurses, in driving evidence‐based clinical practice and encouraging efficient teamwork culture and communication (Proops 2019). Teamwork culture and communication in the operating room particularly have a colossal effect on patient outcomes, including the risk of developing SSIs (Dellinger 2016). Implementation strategies that address barriers in each contributing factor found in this review are believed to help guideline uptake in acute care settings (Sheridan et al. 2020). Research that explores priority barriers that are important and feasible to address from clinicians' and patients' views is imperative, as various barriers can inhibit the implementation of clinical interventions (Craig et al. 2017). Further research is required to identify priority barriers and facilitators to execute SSI prevention and management guidelines to develop a stakeholder‐tailored intervention.

### 6.1 Strengths and Limitations

The five themes constructed in this review provide a broad understanding of factors contributing to the uptake and implementation of SSI prevention and management guidelines, incorporating both clinicians and patients' perspectives. This supports the development of comprehensive, evidence‐informed interventions to improve the prevention and management of SSIs within acute care hospital settings.

However, this review does have some limitations. Only peer‐reviewed articles published in English were included, which could have led to relevant non‐English evidence being excluded. Also, as this review focuses on the acute care setting of a hospital, it provides a context‐specific comprehension of barriers and facilitators to implementing SSI prevention and management guidelines to inform intervention development. Therefore, critical reflection on the contextual adaptability and dependability of the results should be taken into consideration (Bach‐Mortensen and Verboom 2020), which may not be generalisable in other healthcare settings. The methodological rigour of some of the qualitative studies, which had indistinct data collection methods, and quantitative studies, which evidenced problems with the target population representativeness and non‐response bias risk, is another limitation of this review. Further research with high‐quality research methodologies is needed to explore clinicians' and patients' experiences, perceptions, barriers and facilitators for preventing and managing SSIs in acute care.

In some of the articles included in our review the term ‘guidelines’ was used without specific clarification on whether they were international, national, local or a mixture of these. Therefore, we were unable to provide more nuanced analytical detail within this review without making assumptions about what the original authors intended. Consequently, for the purpose of this review, we interpreted guidelines as any formal document designed to inform and guide clinical practice in SSI prevention or management.

## 7 Conclusion

The five themes constructed from this review highlight the significance of providing up‐to‐date evidence‐based guidelines, clinician adherence to these guidelines, the need to address knowledge deficits about SSI prevention to reduce the risk of inconsistent clinical practice, the importance of collaborative interdisciplinary and patient‐provider relationships in SSI management and the significance of infection surveillance in improving patient safety and quality of life. It is important to design and evaluate interventions that address the barriers identified in this review to improve the uptake and correct use of SSI prevention and management guidelines.

## Author Contributions

**Eliza Humphrey:** conceptualisation, data curation, formal analysis, funding acquisition, methodology, writing – original draft, writing – review and editing. **Adam Burston:** conceptualisation, data curation, formal analysis, methodology, supervision, writing – review and editing. **Elizabeth McInnes:** conceptualisation, data curation, review and editing. **Heilok Cheng:** data curation, formal analysis. **Mika Musgrave‐Takeda:** data curation, formal analysis. **Ching Shan Wan:** conceptualisation, data curation, formal analysis, funding acquisition, methodology, project administration, supervision, writing – review and editing.

## Conflicts of Interest

The authors declare no conflicts of interest.

## Supporting information


Data S1.

Data S2.

## Data Availability

Data sharing is not applicable to this article as no new data were created or analyzed in this study.
