# Supplementary material for: Clinicians' and Patients' Experiences and Perceptions on the Prevention and Management of Surgical Site Infections: A Mixed‐Methods Systematic Review
Source: J Clin Nurs. 2024 Nov 22;34(1):24–48. doi: 10.1111/jocn.17443 (PMC11655432; doi:10.1111/jocn.17443)
Supplement: Supplementary file 1 — Data S1. [file JOCN-34-24-s001.docx]

**Supplementary file 1: Full search strategy for all databases**

1. **Medline**

| S1 | \| TI (hospital* or inpatient*or outpatient* or ward* or "acute care*" or "acute setting*" or subacute* or rehab* or "secondary care*" or "tertiary care*") OR AB (hospital* or inpatient* or outpatient* or ward* or "acute care* "or "acute setting* " or subacute* or rehab* or "secondary care*" or "tertiary care*") \| \| --- \| |
| --- | --- | --- |
| S2 | TI (“surgical*” or “surger*”) N2 (“infect*” or “dehisc*”) OR AB (“surgical*” or “surger*”) N2 (“infect*” or “dehisc*” or “disruption*”) OR AB (“wound* N2 (“infect*” or “dehisc*” or “disruption*”) |
| S3 | (MH "Surgical Wound Infection") |
| S4 | (MH "Social Perception") OR (MH "Perception") OR (MH "Self Concept") OR (MH "Life Change Events") OR (MH "Social Facilitation") OR (MH "Comprehension") OR(MH "Patient Navigation")OR (MH "Health Belief Model") OR (MH "Attitude") OR (MH "Behavior") OR (MH "Health Behavior") OR (MH "Delivery of HealthCare") OR (MH "Health Knowledge, Attitudes, Practice") OR (MH "Attitude to Health") OR (MH "Attitude of Health Personnel") |
| S5 | TI (view* or perspective*or experience* or perception* or barrier* or challeng* or facilitator* or understand* or enabler*or belief* or attitude* or behavio#r*) OR AB (view* or perspective* or experience* or perception* or barrier* or challeng* or facilitator* or understand* or enabler*or belief* or attitude* or behavio#r*) |
| S6 | S2 OR S3 |
| S7 | S4 OR S5 |
| S8 | S1 AND S6 AND S7 |
| S9 | S1 AND S6 AND S7 [Narrow by Language: English AND Limit Date of Publication from 01/01/2009] |

1. **EMBASE**

| 1 | (hospital* or inpatient* or outpatient* or ward* or "acute care*" or "acute setting*" or subacute* or rehab* or "secondary care*" or "tertiary care*").ti. |
| --- | --- |
| 2 | (hospital* or inpatient* or outpatient* or ward* or "acute care*" or "acute setting*" or subacute* or rehab* or "secondary care*" or "tertiary care*").ab. |
| 3 | 1 or 2 |
| 4 | (“surgical*” or “surger*”) adj2 (“infect*” or “dehisc*”) ti. |
| 5 | (“surgical*” or “surger*”) adj2 (“infect*” or “dehisc*”) ab. |
| 6 | 4 or 5 or 6 |
| 7 | (view* or perspective* or experience* or perception* or barrier* or challeng* or facilitator* or understand* or enabler* or belief* or attitude* or behavio#r*).ti. |
| 8 | (view* or perspective* or experience* or perception* or barrier* or challeng* or facilitator* or understand* or enabler* or belief* or attitude* or behavio#r*).ab. |
| 9 | perception/ or self concept/ or life event/ or social behavior/ or comprehension/ or patient care/ or attitude to health/ or Health Belief Model/ or health behavior/ or health personnel attitude/ or attitude/ or behavior/ or health care delivery/ |
| 10 | 8 or 9 or 10 |
| 11 | 3 and 7 and 11 |
| 12 | limit 12 to (english language and embase and yr="2009 -Current") |

1. **CINAHL**

| S1 | \| TI (hospital* or inpatient*or outpatient* or ward* or "acute care*" or "acute setting*" or subacute* or rehab* or "secondary care*" or "tertiary care*") OR AB (hospital* or inpatient* or outpatient* or ward* or "acute care* "or "acute setting* " or subacute* or rehab* or "secondary care*" or "tertiary care*") \| \| --- \| |
| --- | --- | --- |
| S2 | TI (“surgical*” or “surger*”) N2 (“infect*” or “dehisc*”) OR AB (“surgical*” or “surger*”) N2 (“infect*” or “dehisc*”) OR TI (“wound* N2 (“infect*” or “dehisc*”) OR AB (“wound* N2 “infect*” or “dehisc*” or “disruption*”) |
| S3 | (MH "Surgical Wound Infection") |
| S4 | (MH "Social Behavior")OR (MH "Perception")OR (MH "Self Concept")OR (MH "Life Change Events") OR (MH "Patient Navigation") OR(MH "Health Personnel as Patients") OR (MH "Health Belief Model")OR (MH "Attitude") OR(MH "Attitude of Health Personnel") OR (MH "Behavior") OR (MH "Health Behavior") OR(MH "Health Care Delivery") OR (MH "Health Care Delivery,Integrated") OR (MH "Attitude to Health") OR(MH "Health Knowledge") OR (MH "Attitude to Illness") |
| S5 | TI (view* or perspective*or experience* or perception* or barrier* or challeng* or facilitator* or understand* or enabler*or belief* or attitude* or behavio#r* ) OR AB (view* or perspective* or experience* or perception* or barrier* or challeng* or facilitator* or understand* or enabler*or belief* or attitude* or behavio#r* ) |
| S6 | S2 OR S3 |
| S7 | S4 OR S5 |
| S8 | S1 AND S6 AND S7 |
| S9 | S1 AND S6 AND S7 [Narrow by Language: English AND Limit Date of Publication from 01/01/2009] |

1. **PsycINFO**

| S1 | TI (view* or perspective*or experience* or perception* or barrier* or challeng* or facilitator* or understand* or enabler*or belief* or attitude* or behavio#r*) OR AB (view* or perspective* or experience* or perception* or barrier* or challeng* or facilitator* or understand* or enabler*or belief* or attitude* or behavio#r*) |
| --- | --- |
| S2 | DE "Perception" OR DE "Social Perception" OR DE "Self-Concept" OR DE "Self-Perception" ORDE "Life Experiences" OR DE "Life Changes" OR DE "Experiences(Events)" OR DE "Social Facilitation" OR DE "Comprehension" OR DE "Health Belief Model" OR DE "Behavior" OR DE "Health Care Seeking Behavior" OR DE "Health Knowledge" OR DE "Health Risk Behavior" OR DE "Health Personnel Attitudes" ORDE "Health Attitudes" OR DE "Health Care Delivery" |
| S3 | TI (“surgical*” OR “surger*”) N2 (“infect*” OR “dehisc*”) OR AB (“surgical*” or “surger*”) N2 (“infect*” or “dehisc*”) OR TI (“wound* N2 (“infect*” or “dehisc*” or “disruption*”) |
| S4 | (DE "Surgical Wound Infection") |
| S5 | TI ( hospital* or inpatient*or outpatient* or ward* or "acute care*" or "acute setting*" or subacute* or rehab* or "secondary care*" or "tertiary care*") OR AB ( hospital* or inpatient* or outpatient* or ward* or "acute care* "or "acute setting* " or subacute* or rehab* or "secondary care*" or "tertiary care*") |
| S6 | S1 OR S2 |
| S7 | S3 OR S4 |
| S8 | S5 AND S6 AND S7 |
| S9 | S5 AND S6 AND S7 [Narrow by Language: English AND Limit Date of Publication from 01/01/2009] |

1. **Cochrane Central Library**

| #1 | ((hospital* or inpatient* or outpatient* or ward* or "acute care*" or "acute setting*" or subacute* or rehab* or "secondary care*" or "tertiary care*")):ti,ab,kw (Word variations have been searched) |
| --- | --- |
| #2 | ((“surgical*” or “surger*”) NEAR/2 (“infect*” or “dehisc*”)):ti, ab, kw (Word variations have been searched) |
| #3 | MeSH descriptor: [Surgical wound infection] this term only |
| #4 | #2 or #3 |
| #5 | ((view* or perspective* or experience* or perception* or barrier* or challeng* or facilitator* or understand* or enabler* or belief* or attitude* or behavio#r*)):ti,ab,kw (Word variations have been searched) |
| #6 | MeSH descriptor: [Social Perception] this term only |
| #7 | MeSH descriptor: [Perception] this term only |
| #8 | MeSH descriptor: [Self Concept] this term only |
| #9 | MeSH descriptor: [Life Change Events] this term only |
| #10 | MeSH descriptor: [Social Facilitation] this term only |
| #11 | MeSH descriptor: [Comprehension] this term only |
| #12 | MeSH descriptor: [Patient Navigation] this term only |
| #13 | MeSH descriptor: [Health Belief Model] this term only |
| #14 | MeSH descriptor: [Attitude] this term only |
| #15 | MeSH descriptor: [Health Behavior] this term only |
| #16 | MeSH descriptor: [Delivery of Health Care] this term only |
| #17 | MeSH descriptor: [Health Knowledge, Attitudes, Practice] this term only |
| #18 | MeSH descriptor: [Attitude to Health] this term only |
| #19 | MeSH descriptor: [Attitude of Health Personnel] this term only |
| #20 | #5 or #6 or #7 or #8 or #9 or #10 or #11 or #12 or #13 or #14 or #15 or #16 or #17 or #18 or #19 |
| #21 | #1 and #4 and #20 with Cochrane Library publication date Between Jan 2009 and May 2021 |

**Supplementary file 2: Quality assessment of included studies using the Mixed-Methods Appraisal Tool, stratified by study design**

| **Author, year** | **Question 1** | **Question 2** | **Question 3** | **Question 4** | **Question 5** |
| --- | --- | --- | --- | --- | --- |
| **Qualitative** |  |  |  |  |  |
| Brown 2014 | Yes | Can't tell | Yes | Yes | Yes |
| Charani 2017 | Yes | Yes | Yes | Yes | Yes |
| Clack 2019 | Yes | Yes | Yes | Yes | Yes |
| Gagliardi 2009 | Yes | Yes | Yes | Can't tell | Yes |
| Gillespie 2012 | Yes | Yes | Yes | Can't tell | Yes |
| Gelhorn 2018 | Yes | Yes | Can't tell | Yes | Yes |
| Ierano 2019 | Yes | Yes | Yes | Yes | Yes |
| Kasatpibal 2018 | Yes | No | Yes | Yes | Yes |
| Larsson 2023 | Yes | Yes | Yes | Yes | Yes |
| Lin 2019 | Yes | Yes | Yes | Yes | Yes |
| Mmari 2021 | Yes | Yes | Yes | Yes | Yes |
| Mottram 2011 | Yes | Yes | Yes | Yes | Yes |
| Tanner 2012 | Yes | Can't tell | No | Yes | Yes |
| Tanner 2013 | Yes | Yes | Yes | Yes | Yes |
| Troughton 2019 | Yes | Can't tell | Yes | Yes | Yes |
| Vieirade Souza 2020 | Yes | No | No | Yes | Can't tell |
| Walker 2020 | Yes | Yes | Yes | Yes | Yes |
| **Quantitative** |  |  |  |  |  |
| Accardi 2017 | Yes | Yes | No | No | Yes |
| Ahmed 2019 | Yes | Can't tell | Yes | Yes | Yes |
| Altaweli 2023 | Yes | Yes | Yes | Can't tell | Yes |
| Anderson 2013 | Can't tell | Can't tell | Yes | Yes | Can't tell |
| Badia 2020 | Yes | Can't tell | Yes | Can't tell | Can't tell |
| Badia 2020 | Yes | Can't tell | Can't tell | No | Can't tell |
| Balodimou 2018 | Can't tell | No | No | Can't tell | Yes |
| Cooper 2019 | Can't tell | Can't tell | Can't tell | Yes | Can't tell |
| Ding 2017 | Yes | Can't tell | Yes | Yes | Yes |
| Eskicioglu 2012 | Yes | Can't tell | Can't tell | No | Yes |
| Ghuman 2021 | Yes | Can’t tell | Can’t tell | No | Can’t tell |
| Gillespie 2014 | Yes | Can't tell | Yes | No | Yes |
| Merle 2011 | Can't tell | No | No | Yes | Can't tell |
| Moran 2018 | Yes | No | Can't tell | No | Yes |
| Pucher 2014 | Can't tell | Can't tell | Can't tell | Yes | Yes |
| Ryu 2020 | Yes | Can't tell | No | Can't tell | Can't tell |
| **Mixed-methods** |  |  |  |  |  |
| Do 2021 | Yes | Yes | Yes | Can't tell | Yes |
| Lin 2020 | Yes | Can't tell | Yes | Yes | Yes |
| Sickder 2017 | Can't tell | Can't tell | No | Can't tell | Can't tell |

The Mixed Methods Appraisal Tool used in this mixed-methods systematic review was found in: Hong QN, Pluye P, Fàbregues S, Bartlett G, Boardman F, Cargo M, Dagenais P, Gagnon M-P, Griffiths F, Nicolau B, O’Cathain A, Rousseau M-C, Vedel I, 2018. Mixed Methods Appraisal Tool (MMAT). Canadian Intellectual Property Office, Canada.

**Supplementary file 3: Quality assessment of the included quality improvement study**

| **Quality Improvement** | **Question 1** | **Question 2** | **Question 3** | **Question 4** |
| --- | --- | --- | --- | --- |
| Ackers 2020 | Met | Met | Met | Met |
|  | **Question 5** | **Question 6** | **Question 7** | **Question 8** |
|  | Met | Not met | Met | Met |
|  | **Question 9** | **Question 10** | **Question 11** | **Question 12** |
|  | Met | Met | Not met | Met |
|  | **Question 13** | **Question 14** | **Question 15** | **Question 16** |
|  | Met | Met | Not met | Not met |

The Quality Improvement Quality Criteria Set Tool used in this mixed-methods systematic review was found in: Hempel S, Shekelle PG, Liu JL, Sherwood Danz M, Foy R, Lim YW, Motala A, Rubenstein LV, 2015. Development of the Quality Improvement Minimum Quality Criteria Set (QI-MQCS). British Medical Journal Quality & Safety.
